# The Transactions of the Esculapian Society of the Wabash Valley, at Its Annual Session Held at Tuscola, Illinois, October 26-7, 1870

**Published:** 1871-04

**Authors:** Wm. M. Chambers, Wm. E. Henry

**Affiliations:** Pres., Charlestown, Ill.; Sec., Mattoon, Ill.


### THE TRANSACTIONS OF THE ESCULAPIAN SOCIETY OF THE WABASH VALLEY, AT ITS ANNUAL SESSION HELD AT TUSCOLA, ILLINOIS, OCTOBER 26-7, 1870.

The meeting was not well attended, and reports from only a few of those appointed chairmen of committees were heard from. Dr. Albin, of Neoga, although present, failed to make a report on surgery, and but for a few cases reported, this department would have been a complete failure. One of special interest, from Dr. L. J. Willien, a case of prostatic abscess. Thos. S., aged 29; tall, rather stooped; of good constitution; well-digger, was taken sick in July, with severe pains in the region of the bladder, and stranguary; was treated empirically for a while, and had received injections of acetate of lead, zinc, and chlo. iron into the bladder—still grew worse till I saw him first, on September 4th, 1870. The following symptoms presented: eyes sunken, expressive of pain; face pinched and covered with a cold copious perspiration; mouth dry, tongue furred, great thirst, with anorexia; skin hot and clammy, with cold sweats; pulse small, 100 per minute; emaciated; severe pain in region of perineum, which was hot and swollen, and over the bladder, which was much distended, great restlessness.

Diagnosis obscure, first thought it a case of rheumatic cystitis, on account of the trade the patient followed; yet the greatest pain being near the neck of the bladder, and extending along the urethral canal and spermatic cords, with considerable swelling of these organs, led me to seat the disease in the prostate gland, which I fully convinced myself of by introducing a catheter, which passed the gland with difficulty, but after entering the bladder gave no pain, but only relieved it of a large amount of urine, with mucus detritis.

TREATMENT.—Infusion of flax-seed, Oss, tinct. opii, ʒi, injected into the bladder every half-hour; the patient felt relieved and in a short time micturated without the bougie; narcotic poultices to the perineum; turpentine frictions over pubic region; internally,

| | | |
|---|---|---|
| ℞. | Potass. Acetatis, | ℥ss. |
| | Wine Cholch Sem., | f. ℥ss. |
| | Tinct. Opii Comporata, | f. ℥i. |
| | Aqua Mentha Pip, | f. ℥ij. |
| | Syr. Tolu, | f. ℥j. |

Mix; take one teaspoonful every four hours; watermelon seed infusion to drink.

6 P.M., removed one pint urine, very ammoniacal, with few flakes of pus; the above injection repeated, and treatment continued.

5th, 8 A.M., slept all night; micturated twice; pulse soft and regular, 75; expression of face calm; some appetite; no pain in bladder or perineum.

11 A.M., severe chill, lasting nearly one hour, with pain in neck of bladder and perineum, with swelling and heat, followed with profuse perspiration and free evacuation of bowels; micturation impossible, catheter introduced.

8 P.M., fever less; perspiration profuse; pulse small 120; tongue coated in centre, edges very red; severe tension of the perineum, and swelling of the scrotum, urethra, and penis; left testis very painful. Narcotic poultice to the perineum; sulph. quinine, grs. iv, every three hours.

6th, 8 A.M., urine high-colored; alkaline mixed with pus; fever continues.

℞. Unguent Hydrag.,-------------------d.s. ℥ss.

Ext. Belladonna,--------------------- gr. v.

" Strammon.,-------------------------gr. v.

Pulv. Camphor,----------------------- ℈ij.

" Ammon. Mur.,-------------------- ʒj. M.

Ft. unguent to be rubbed on perineum and over urethra every three hours.

℞. Quinine Sulph.,-----------------------℈j.

Pulv. Opii,-------------------------gr. x.

" Camphor,------------------------gr. xxi.

Mix; divide 20 powders; one every three hours. Urinary secretion being established, acetate of potassa is suppressed, and the following substituted:

℞. Spts. Terebinth,--------------------------ʒj.

Musc. Acacia,-----------------------------℥j.

Tinct. Opii,-------------------------------ʒij.

Syr. Simple,-------------------------------℥j.

Aqua Mentha Pip,--------------------------℥ij. M.

Cochlea magna tria horis.

7th, 8 A.M., patient rests; pulse 90 and regular; less thirst; two pints of urine passed during the night by aid of catheter; local symptoms as above; treatment same.

8 P.M., ut supra; urine mixed with pus.

8th, 8 A.M., pulse 78; other symptoms better; exploration per rectum; engorgement of prostate gland, very hot and painful, its left and posterior side distended and fluctuating; above treatment continued.

7:30 P.M., pulse 80, regular; skin soft and moist; perineum left side much swollen, with a throbbing pain; treatment ut supra.

9th, 8 A.M., bowels moved; urine drawn by catheter; local symptoms the same.

8 P.M., perineum as before; made a free incision; large quantity of pus and gas escaped; continued poultices, and gave, internally, in place of above,

℞. Tinct. Cinchonæ Comp., ................ f. ℥iv.

ʒiss three times a day.

10th, 9. A.M., symptoms all better; passes urine without catheter; matter continues to discharge itself; incision washed out with carbolic acid water.

11th, 9 A.M., patient much better; appetite good; exploration per rectum reveals an entire subsidence of past symptoms; same treatment continued. Patient made a hasty recovery, and was walking about, most well September 20th.

Dr. C. B. Cannon's report on midwifery was full, and some cases, with treatment, elicited a lengthy debate. One of abnormal presentation and turning, with hour-glass contraction (labor lasting 55 minutes); retained placenta with hemorrhage. Dr. W. Brinton had reported a case in which there was rupture of the membranes and escape of the waters some *three weeks* before the delivery of a living child. This case caused much doubting, and, although it came from so reliable a source, some members were still inclined to discredit it, presuming there must have been some abnormal condition which led the observer into error, since the child was alive and healthy. The report and debate lasted most of the first day.

Dr. Walston, of Paris, Illinois, failed to report on indigenous botany.

Dr. Johnson, of New Goshen, Indiana, being absent, there was no report on epidemics.

Dr. L. L. Todd, of Paris, Illinois, also failed to report on practical medicine. Indeed, all but one appointed failed to "come to time;" however, the meeting was not without benefit, as every one present felt a deep interest in the cases reported.

Dr. M. W. Wilcox read a paper on "Criminal Abortion"—"Slaughter of the Innocents," and one on the "Current Medical Literature of the Day." The first was in defence of the profession proper, laying the blame on a *non-professional* class or *empirics*, and society, as well as the law-making bodies of our country, for its permission. This paper was full of sarcasm, wit, and humor, with which its author is at all times peculiarly fortified.

The second paper was one on which the practical part of the profession would all agree, we think. Its author designed to show the unreliability of many of our text-books, as well as a great part of what was written for medical journals at the present day; and a great many practitioners too implicitly relied on what they read, which resulted, not only in great dissatisfaction to themselves, but was often fatal to their patients. He thought it doubtful whether Aitken or Bennett could describe and lay down a course of treatment of a special disease common to England and the State of Illinois, or, in other words, that they could treat as successfully in some localities of Illinois as they could the same disease at home, in England, at least, he thought the *modus operandi* of calomel in Illinois, was diametrically opposite to Dr. Bennett's in England. He thought there were too many writers by profession whose faculty of imagination became heightened or too exquisite for practical men. He did not wish to be understood as opposed to what was new, but that new ideas, which were only ideas, should be viewed with caution, especially when they may come in opposition to vital force.

The public address was also delivered by Dr. M. W. Wilcox, on his retiring from the presidency of the Society, which was conceded by all members present to have been the best adapted for a public audience they ever listened to.

Dr. J. H. Apperson, of Tuscola, on account of sickness, did not prepare his report on "The Sources of the Gastric Juice," and was continued for the next meeting. Dr. A. seems to take issue with the rest of his brethren, in the Society at least, but has a great deal of modesty in opposing his own favored views to a universally received theory; yet, while he was aware that theories no less cherished and generally received had fallen, while new ones had sprung up. The opinion of the doctor is, simply that the glands of the mouth, the saliva, furnish the *warm and only fluid necessary in digestion*, and that the *stomach secretes only mucus.* It is unnecessary to state that nearly every member of the Society was opposed, in a warm discussion, to Dr. A., yet they requested him to reduce his arguments to writing at the Society's next semi-annual meeting, when it is hoped, if there is something new yet in this direction, it may come to light. Dr. Apperson is an old and able physician, and his views will be doubtless interesting.

The Society then elected its officers for the ensuing year: Dr. Wm. M. Chambers, of Charlestown, Ill., was unanimously made President; Dr. L. J. Willien, of Effingham, Vice-President; Wm. E. Henry, Secretary and Treasurer. Delegates to American Medical Association: Drs. Wilcox, Chambers, Cannon, TenBrook, Swafford, Steel, and Henry. Drs. Willien, Miller, Reat, Lacron, Brinton, Apperson, and Denning, as delegates to State Society.

After a two days' meeting, during which time its members were well entertained by the resident physicians of Tuscola, the Society adjourned to meet at Effingham, Illinois, on May 25th, 1871.

WM. M. CHAMBERS, M.D. *Pres.*,

Charlestown, Ill.

WM. E. HENRY, M.D., *Sec.*,

Mattoon, Ill.

